# Rapid and sensitive detection of *E. coli* O157:H7 by lateral flow immunoassay and silver enhancement

**DOI:** 10.1007/s00604-023-05834-8

**Published:** 2023-06-19

**Authors:** Shayesteh Bazsefidpar, Esther Serrano-Pertierra, Gemma Gutiérrez, Alberto Sánchez Calvo, María Matos, María Carmen Blanco-López

**Affiliations:** 1 Department of Physical and Analytical Chemistry & Institute of Biotechnology of Asturias, University of Oviedo, c/Julián Clavería 8, 33006 Oviedo, Spain; 2 Department of Chemical and Environmental Engineering & Institute of Biotechnology of Asturias, University of Oviedo, Oviedo, Spain

**Keywords:** *E. coli* O157:H7, Silver enhancement, PEG polymer, Sandwich-LFIA, Food analysis, Foodborne pathogens

## Abstract

**Abstract:**

The aim of this study was to develop a sensitive lateral flow immunoassay (LFIA) for the rapid detection of *Escherichia coli* (*E. coli*) O157:H7, a pathogen contributor to diseases and fatalities worldwide. Au nanoparticles with high stability, uniform size, and shape were synthesized and coated with heterobifunctional PEG polymer with carboxyl groups, and they were bioconjugated to be used as label in sandwich-LFIA. Then, a silver enhancement strategy was developed as an accessible, rapid, and cost-effective approach for signal amplification to reduce the limit of detection (LOD). The optimal results were achieved when a solution of silver nitrate and hydroquinone/citrate buffer was added to the strips for 4 min. This led to a decrease in the visual LOD from 2 × 10^6^ (CFU mL^−1^) to 2 × 10^3^ (CFU mL^−1^), resulting in a threefold improvement in sensitivity compared to the conventional LFIA system. The specificity of the system was evaluated by using non-target bacteria (*E. coli* BL21 and *E. coli* T515) and its reliability was determined by testing commercial food samples (milk, tap water, and orange juice), demonstrating its effectiveness for quickly detecting pathogenic bacteria in food products.

**Graphical Abstract:**

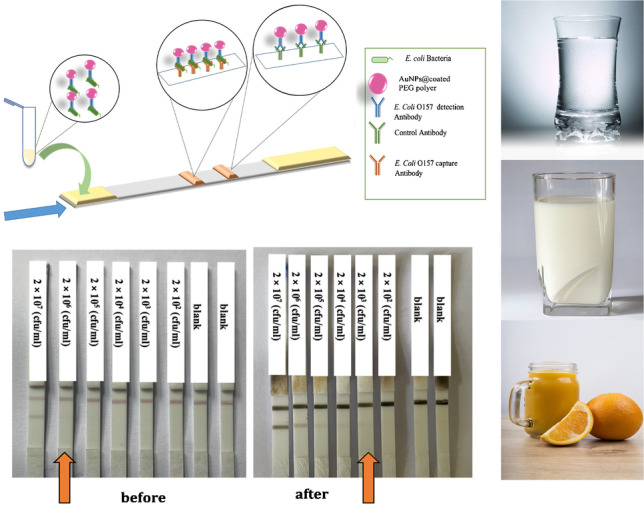

**Supplementary Information:**

The online version contains supplementary material available at 10.1007/s00604-023-05834-8.

## Introduction

*E. coli* O157:H7 is a serotype of the bacterial species *E. coli*, and it belongs to a subset of Shiga toxin producers [1]. It causes severe health problems, usually through consuming contaminated or uncooked food, such as raw milk or ground beef that has not been adequately cooked. According to the World Health Organization (WHO), *E. coli* O157:H7 is a bacterial strain that can cause severe foodborne illness, including bloody diarrhea and kidney failure. Therefore, many countries have established regulations regarding the allowable concentration of *E. coli* O157:H7 in food to protect public health [2, 3]. In the USA, the Food and Drug Administration has set a zero tolerance policy for *E. coli* O157:H7 in certain foods, including ground beef. This means that any detectable amount of *E. coli* O157:H7 in these foods is illegal and can result in a product recall or enforcement action (https://www.usda.gov/topics/health-and-safety). The European Union has established regulatory limits for *E. coli* O157:H7 in various food categories, such as raw beef and beef products, sprouts, and milk, among others. For example, the maximum allowable concentration of *E. coli* O157:H7 in raw beef is 100 CFU g^−1^ (colony-forming units per gram), while the maximum allowable concentration in sprouts is 10 CFU g^−1^ [3]. Therefore, there is a strong need for quicker methods to detect pathogens to ensure public health and safety.

The available microbiological culture detection methods often require about 18–72 h to reach the suitable concentration for quantification. Immunological methods and techniques such as polymerase chain reaction and enzyme-linked immunosorbent assay require expensive equipment, laborious protocol, and technical expertise [4, 5]. It is therefore crucial to develop a fast, simple, straightforward, and sensitive technique for early detection of pathogens at low levels.

LFIAs as point-of-care diagnostic tools are cost-effective, robust, rapid, and user-friendly with diverse applications in various fields such as agriculture, food safety, environmental science, and medicine [6, 7]. However, LFIAs have certain drawbacks that can pose a challenge for researchers. These include limitations in terms of their sensitivity, non-specific binding, and inability to offer quantitative results [8]. Maintaining consistency and reliability in LFIAs is crucial, and this can be influenced by several factors, including the assay’s sensitivity and specificity, sample matrix, handling of assay components, and variability of instruments and operators [9].

Numerous studies have aimed to enhance the reliability and sensitivity of LFIAs by utilizing various labeling methods, including colloidal metal nanoparticles [10], magnetic beads [11], and quantum dots [10], along with signal amplification techniques like electrochemistry [12], enzyme catalysis [13], and silver/gold enhancement [14, 15]. Among these techniques, silver/gold enhancement offers several advantages over other techniques for enhancing sensitivity of LFIAs including high sensitivity, simplicity, rapid results, cost-effectiveness, and portability [14]. Silver staining protocols for enhanced protein detection in electrophoresis gels or in electron microscopy are a source of inspiration for LFIA systems. Several methods have been developed for toxins and biomarkers [7], and they have been applied on commercial AuNPs. The silver/gold enhancement technique involves depositing a thin layer of silver on top of the AuNPs located on the test line of an LFIA strip [14]. This enhances the signal generated by the AuNPs, leading to a higher sensitivity of the assay and the ability to detect low levels of the target analyte in LFIAs. The silver deposition on the surface of AuNPs is carried out by mixing silver salts such as nitrate or acetate with a reducing agent including hydroquinone (1,4-dihydroxybenzene), catechol (1,2-dihydroxybenzene), and resorcinol (1,3-dihydroxybenzene) [16, 17] which causes a significant color change from red to black [18]. Our research group has compared several techniques to deposit silver on the AuNPs situated on the test line of the LFIA strip, including the immersion assay, sandwich immunochromatographic assay, and conjugated pad modified with silver [14]. We found that the highest signal was produced using the immersion method, which involved a 1:1 solution of silver nitrate and hydroquinone/citrate buffer. Using this approach, we were able to improve the sensitivity of the system one order of magnitude. In other work, Panferov et al. [19] developed a fast quality control method to lower the limit of detection (LOD) for *R. solanacearum* using silver-enhanced LFIA. They utilized fiberglass membranes pre-absorbed with silver lactate and hydroquinone, which were placed on the analytical zone, and added a drop of silver lactate for silver enhancement. The study found that this approach improved 10 times the sensitivity of the system.

Apart from the advantages of this technique, the use of silver enhancement in LFIA has some limitations and considerations, such as potential interference from sample matrix components, or the non-specific binding of silver to non-target molecules. These aspects should be considered for optimization of the assay.

Spherical AuNPs are the most frequently nanomaterials used as label in LFIAs. These materials are popular due to their favorable physical and chemical properties, such as their optical properties, ability to be easily functionalized and stabilized, strong affinity for biomolecules, non-toxicity, affordability, and quantum and plasmonic properties, which also have potential catalytic applications [20, 21]. However, the non-specific binding of the AuNPs on the test line is a frequent problem during LFIA development, and it is important to minimize false positive results. By reducing nonspecific binding, the specificity and sensitivity of the test can be improved, leading to more accurate results [22]. One common approach is to use biocompatible and hydrophilic coatings, such as polyethylene glycol (PEG), on the surface of the nanoparticles to prevent the adsorption of proteins and other molecules. Additionally, PEGylation of AuNPs improves their dispersion and stability, prevents aggregation and denaturation of the antibodies, and minimizes the non-specific binding which is a critical consideration when designing labels for LFIA systems [21, 23, 24].

In this study, a sandwich-type LFIA was developed for rapid and specific detection of *E. coli* O157:H7. AuNPs with an average size of 40 nm were synthesized and functionalized with heterobifunctional (SH-PEG-COOH, 5KDa) with carboxyl groups for bioconjugation with the antibody. A silver enhancement technique was developed for signal amplification. Quantitative detection was achieved by analysis of the signal intensity on the test line or the colorimetric signal after the silver enhancement through a portable strip reader. The specificity was investigated by using non-target bacteria (*E. coli* BL21 and *E. coli* T515) and to confirm the effectiveness of the developed assay, the strips were applied to detect *E. coli* O157:H7 in three different types of food samples: milk, drinking water, and fruit juice.

## Experimental

### Reagents and instruments for the immunoassay

Mouse monoclonal antibodies to *Escherichia coli* O157:H7 (MBS568290 and MBS568193) were purchased from Mybiosource (San Diego, CA, USA).

Goat anti-mouse IgG, N-hydroxysuccinimide (NHS), 1-ethyl-3-[3-dimethylaminopropyl]–carbodiimide hydrochloride (EDC), 2-(N-morpholino)ethanesulfonic acid (MES), bovine serum albumin (BSA), phosphate-buffered saline (PBS), silver nitrate (AgNO_3_), hydroquinone (C_6_H_4_-1,4-(OH)_2_), gold(III) chloride hydrate (HAuCl_4_), and sodium citrate tribasic dihydrate (C_6_H_5_Na_3_O_7_·2H_2_O) were obtained from Sigma-Aldrich (St. Louis, MO, USA). Sodium dodecyl sulfate (SDS) (C1_2_H_2_5NaO_4_S) was purchased from PanReac AppliChem (Barcelona, Spain).

HS-C_2_H_4_-CONH-PEG-C_3_H_6_-COOH, MW = 5000 g mol^−1^ (4900 Da), and CH_3_O-PEG-NH_2_, MW = 725–850 Da were purchased from RAPP POLYMER (Tuebingen, Germany). All the reagents were prepared using Milli-Q ultrapure water (resistivity 18.2 MΩ·cm at 25 °C), unless otherwise stated.

An IsoFlow reagent dispensing system (ImageneTechnology, USA) was used to dispense the detection lines (dispense rate 0.100μL mm^−1^) and the strips were cut with a guillotine Fellowes Gamma (Spain).

A portable strip reader ESE Quant LR3 lateral flow system (Qiagen Inc., Germany) was used to quantify the intensity of the test line by reflectance measurements.

### Bacterial strains

*E. coli* O157:H7, *E. coli* BL21, and *E. coli* CEC T515 were cultured in 50 ml of Tryptic Soy Broth medium at 37°C and agitation speed of 240 rpm, overnight. The culture media was centrifuged and resuspended in 5ml of PBS. In order to inactivate the *E. coli* O157:H7 for safe handling, the cells were heated at 100°C for 15min. Samples were frozen until further use.

### Synthesis of citrate-AuNPs and PEGylation

The colloidal AuNPs coated with PEG polymer were synthesized through a two-step process that involved: (i) synthesizing the AuNPs using a seeded growth method and (ii) subsequent coating with the SH-PEG-COOH 5KDa polymer. To achieve an average nanoparticle size of 40 nm, the ratio of HAuCl_4_ to sodium citrate (1:0.4; 1:2; 1:4) was optimized. In the first step, a seed solution was made by preparing a solution of HAuCl_4_ (77, 51, or 25.4 mg) in 293 ml of Milli-Q water and sodium citrate (33, 99, or 99 mg) in 996.3 μl of Milli-Q water separately. The pH of the HAuCl_4_ solution was set to 5.4±0.5 using a pH meter, and it was then added to a three-neck round-bottomed flask and heated to 100°C with vigorous stirring (900 rpm) and reflux. Upon reaching 100°C, the sodium citrate was quickly injected, and the stirring was continued at that temperature for 90 min. After the reaction was stopped, the mixture was cooled to room temperature and protected from light. The size of the resulting nanoparticles was measured using UV-Vis spectroscopy after cooling.

In the following step, the suspension was stabilized using a heterobifunctional HS-PEG, 5kD via the Au-SH functional groups of the PEG polymer. The synthesized nanoparticles were first concentrated to 10 nM through centrifugation at 10000 rpm for 10 min. Then, 125 μl of SDS 10% and 120 μl of HS-PEG-COOH (458 Da) at 10 mg mL^−1^ were added to the solution and mixed to reach a final volume of 50 ml after the addition of Milli-Q water. Finally, 675 μL of 2 M NaOH was added to the solution, which was covered with aluminum foil and incubated overnight at room temperature with gentle mixing. After incubation, the nanoparticles were centrifuged three times at 10000 rpm for 10 min to purify them, removing any extra PEG polymer and unreacted starting materials. The size and concentration of the nanoparticles were determined by measuring their absorbance.

### Characterization of AuNPs

#### UV-Vis spectroscopy

The spectral analysis was performed in the 200–800 nm range at room temperature with a UV-Vis spectrophotometer (PG Instrument, LTD). The concentration of the AuNPs was calculated with the following equation:

$$A=\varepsilon \times C\times l$$

where *A* is the absorbance at 450 nm, *ε* is the molar absorption coefficient of AuNPs 1.76 × 10^8^ cm M^−1^ or 11.1 ml cm^−1^ mg^−1^ at their localized surface plasmon resonance (LSPR) 450 nm [25, 26], *C* is the molar concentration, and *l* is path length of light traveling in the cuvette.

The size of nanoparticles was determined based on the absorbance at the maximum absorption peak by UV-Vis spectroscopy, following a method reported by Heiss et al. at the literature [25].

#### Transmission electron microscopy

The size and shape of AuNPs were characterized by TEM (JEOL-2000 Ex II TEM (Japan)).

#### Dynamic light scattering

The surface properties and particles size were determined by the ζ-potential value and dynamic light scattering respectively (Zetasizer Nano ZS (Malvern Instruments Ltd., UK).

### Conjugation of AuNPs with E. coli O157:H7 antibody

The AuNPs coated with PEG-COOH were then functionalized with an anti-*E. coli* monoclonal antibody to create the immunoassay. Carbodiimide chemistry was used to activate the carboxyl groups: 1.5 ml of EDC (3 mM in MES 10 mM, pH 6) and 1.5 ml of NHS (7 mM in MES 10 mM, pH 6) were mixed with AuNPs with the concentration of 0.5 mg mL^−1^ for 30 min. To remove the excess of EDC and NHS, the nanoparticles were washed twice with MES buffer 10 mM by centrifugation at 10,000 rpm for 10 min. After centrifugation, *E. coli* detection antibody with different final concentration of 2, 4, 6, and 8 μg mL^−1^ in MES buffer 10 mM, pH 5.5 mixed with nanoparticles for 1.5 h. Then, 500 μl of CH_3_O-PEG-NH_2_ as a blocking agent was added to the solution and incubated at the same operational condition for 2h (concentration of 2.5% of 500 μl of MES buffer 10 mM, pH 5.5). The nanoparticle solution was centrifuged at 10,000 rpm for 10 min and washed once with MES buffer then with HEPES buffer 10 mM, pH 7.4. After removing the supernatant, the mixture was resuspended in 200 μl of stabilizer solution including HEPES buffer 10 mM, pH 7.4, Tween 20 0.1%, and BSA 0.1% and stored at 4°C.

### Lateral flow strip manufacturing and assembly

A sandwich LFIA was carried out for detection of *E. coli* O157 bacteria. The nitrocellulose membrane was incorporated onto a backing card. Then, capture (anti-*E. coli*) and control (goat anti-mouse IgG) antibodies (1 mg mL^−1^) were dispensed on the membrane at a rate of 0.100 μL mm^−1^ for the test and control lines, respectively. The membrane was then dried for 1 h at 37°C. To assemble the strips, the sample pad and the absorbent pad were stuck onto the backing card, with an overlap between them of 2 mm. The complete strip was cut into individual 5-mm-wide dipsticks. For the optical density quantification, a portable reader (ESEQuant, Qiagen) was used.

### Silver enhancement of LFIA test strips

After optimization of the immunoassay, the strips were washed with Milli-Q water. The immersion protocol for silver enhancement was carried out according to a protocol developed in our research group with some modifications [14]. A solution of silver nitrate (0.3%w/v in water) and hydroquinone (3% w/v in 0.5 M citrate buffer pH 4.0) as a reducing agent was prepared and stored at room temperature protected from light. Just before use, the enhancing solution was freshly prepared by 1:1 mixing the two solutions and the volume of 40 μl was dropped onto the test zone in a dark place and left there until the signal appeared. Then, the strips were washed with Mili-Q water to stop the reaction, and they were kept protected from light. All experiments were carried out by triplicate.

### Food sample analysis

To evaluate the applicability of the proposed assay, three real food samples were used: orange juice, liquid milk, and tap water. Orange juice and milk were purchased from a local market. The samples (orange juice and liquid milk) firstly were diluted with PBS buffer (1:10). Then, the diluted samples were spiked with different concentrations of *E. coli* O157 bacteria (2×10^5^, 2×10^3^, 2×10^2^ CFU mL^−1^). Control samples (non-spiked) were also used to investigate the effect of the complex matrix on the proposed sandwich assay and silver enhancement strategy.

## Results and discussion

### Optimization and characterization of synthesis of AuNPs

The optimal size for spherical AuNPs in LFIAs is typically within the range of 20–40 nm. This size range is considered optimal because nanoparticles produce a bright red color after accumulation in the test-line, as a result of the LSPR effect. This way, the line can be detected with naked-eyes or recorded by optical readers for qualitative and quantitative measurement, respectively [27]. AuNPs that are too large may result in aggregation, while smaller AuNPs are less favorable at the LSPR and reduce sensitivity. Hence, it is crucial to have the appropriate size of AuNPs to attain the best results and precision in LFIA [27, 28].

The size of AuNPs is mainly dependent on the concentration of sodium citrate during the synthesis [29, 30]. However, other factors such as the concentration of gold salt, the optimal pH, and temperature during preparation also play a role in determining the final particle size of AuNPs [29]. These factors can all impact the nucleation, growth, and stability of the AuNPs and, therefore, must be carefully considered and controlled to achieve the desired particle size. In this work, spherical AuNPs with an average size of 40 nm were synthesized by the reduction of HAuCl_4_ using citrate as a capping and stabilizing agent. Briefly, the synthesis of spherical AuNPs consists of the reduction of gold by sodium citrate and the stabilization of the colloidal AuNPs in an aqueous suspension [31]. During the synthesis, the citrate forms a larger layer around the colloids and partially blocks their growth. Thus, the final size of the nanoparticles is strongly related to the ratio between HAuCl_4_ and sodium citrate. The pH of the solution also has a dramatic effect on the size, morphology, and stability of AuNPs [20, 32]. The initial pH of HAuCl_4_ solution before adding sodium citrate was set at 5.4±0.5 in all the experiments.

**Fig. 1:**
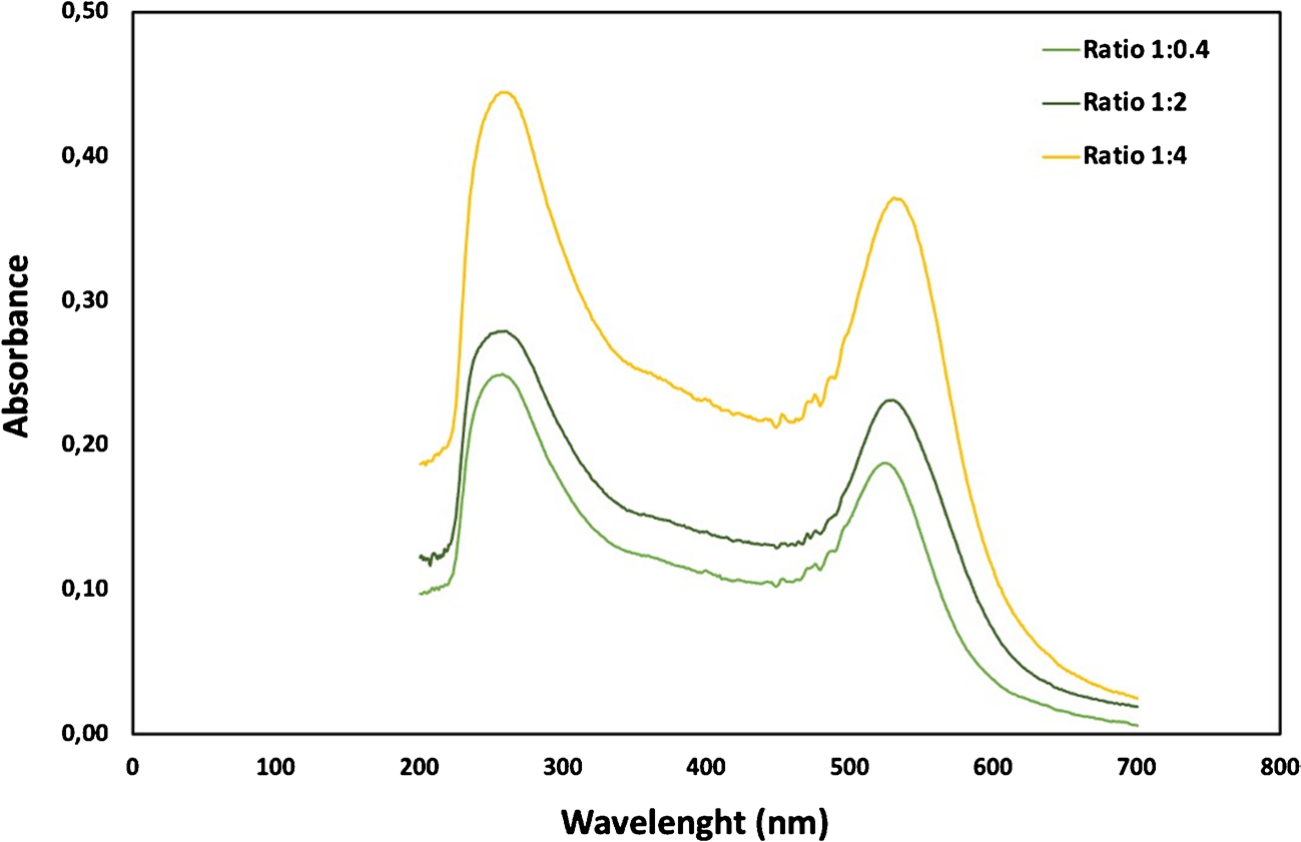
The UV-Vis absorption spectrum of AuNPs prepared with different HAuCl_4_: sodium citrate ratio (1:0.4; 1:2; 1:4) at pH 5.4±0.5, 100°C for 90 min

Figure 1 displays the UV-Vis absorption spectrum of AuNPs that were prepared by using different ratios of HAuCl_4_ to sodium citrate. The results show that the maximum LSPR peak of AuNPs with the ratio of 1:0.4, 1:2, and 1:4 was at 520 nm, 524 nm, and 530 nm, respectively, corresponding to particle sizes of 15 nm, 20 nm, and 40 nm. The data suggests that a higher concentration of sodium citrate leads to larger particle sizes of AuNPs after 90 min of synthesis. In the other work, Ji et al. [33] reported that, by increasing the Na_3_Ct/HAuCl_4_ ratio from 0.7:1 to 3.5:1, the size of synthesized AuNPs decreased. However, a further increasing trend of average size was observed from 3.5: 1 to 28:1, instead of a continuous reduction of the average size. The presence of unreacted citrates could be one possible explanation for the observed increase in the size of AuNPs at high Na_3_Ct/HAuCl_4_ ratio. Citrates are known to have a high affinity for the surface of Au nanoparticles, and excess citrate can form a stable layer on the surface of the nanoparticles, preventing further growth [33, 34].

**Fig. 2:**
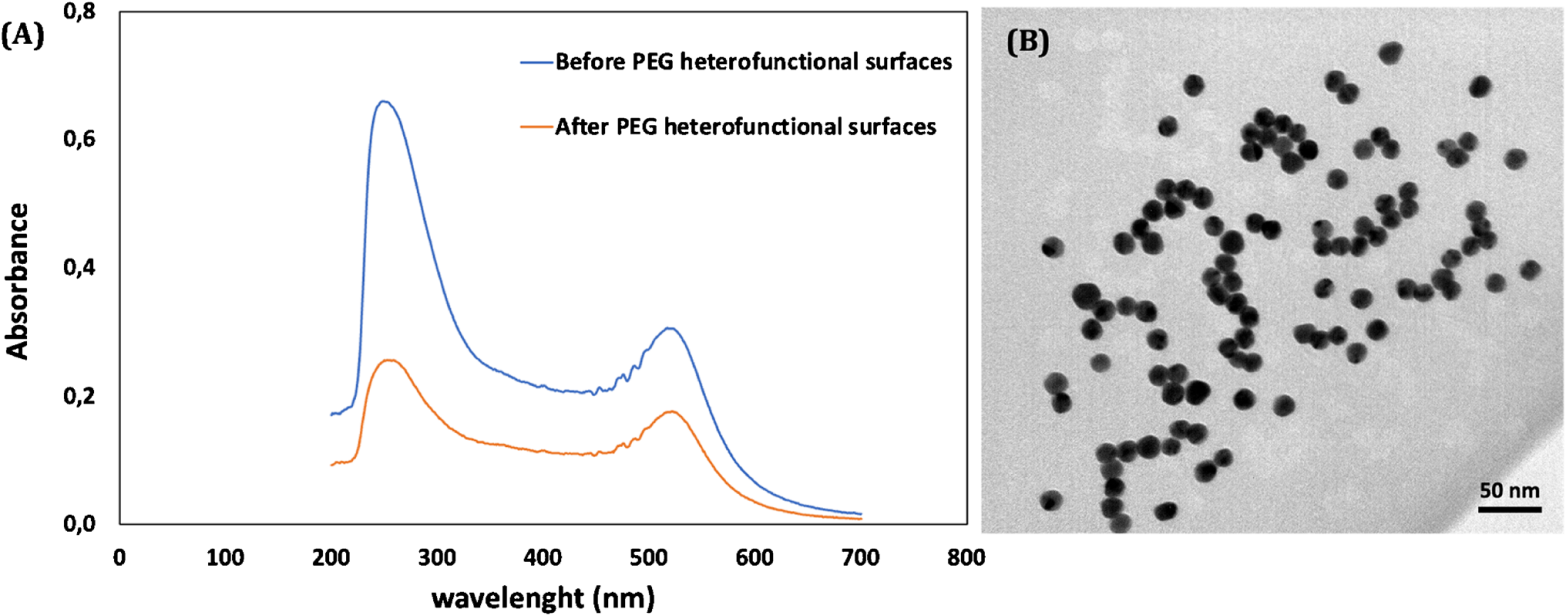
Characterization of the synthesized AuNPs: **A** UV-Vis absorption spectra and **B** transmission electron microscopy (TEM) micrograph of AuNPs

AuNPs with the maximum LSPR peak at 530 nm were considered in this work. They were subsequently coated with heterobifunctional PEG of a length of 5 kDa (SH-PEG-COOH). The concentration of AuNPs was determined using the formula provided in the “UV-Vis spectroscopy” section and the method from reference [25], and subsequently, a fixed value of 10 nM for AuNP PEGylation was set for this work. The PEGylation of AuNPs is essential to avoid aggregation and non-specific adsorption of the nanoparticles [23]. It also provides a suitable surface modification by providing the most adequate chemical groups for covalent binding with several biomolecules. One of the most common approaches to coat plasmonic nanoparticles is by using thiol-terminated derivatives (SH-PEG-COOH) that graft onto the surfaces of NPs covalently. In the case of the synthesis of AuNPs with citrate, by adding thiol-terminated derivatives (SH-PEG-COOH), the citrate ligands progressively exchange the SH-PEG-COOH on the surface of NPs increasing the stability of colloidal solution at high ionic strength solutions [35, 36]. On the other hand, it has been reported in the literature that the addition of SDS helps to improve the dispersion of the nanoparticles and prevent aggregation, while the use of NaOH creates an alkaline environment for coating and functionalizing the nanoparticles [37]. Fig. S1 shows the reaction of synthesized nanoparticles on the strip before and after PEGylation. As it can be seen, the PEGylation of AuNPs effectively eliminates the non-specific binding between the AuNPs and the capture antibody on the test line. Figure 2 displays the characterization of the UV-Vis spectra of the AuNPs both before and after PEGylation, as well as the distribution and size of the nanoparticles, as seen in a TEM micrograph. After the PEGylation of AuNPs, the maximum LSPR peak has only slightly increased (from 530 to 531 nm). Further characterization by means of dynamic light scattering and Z-potential measurements after PEGylation (Fig. S2 also) show a size of 42.9 ± 0.2 nm and a Z-potential of −31 ± 2 mV, respectively. These results confirm the successful PEGylation of the nanoparticles.

### Development of a sandwich lateral flow immunoassay method

The first step of development of the immunoassay was the optimization of the amount of *E. coli* O157 detection antibody for conjugation with AuNPs. Different total concentrations of (2, 4, 6, and 8 μg mL^−1^ of antibody) were mixed with 0.5 mg mL^−1^ of AuNPs. The minimum concentration was set by analyzing the signal intensity generated on the testing line using a portable strip reader (Fig. S3). The running buffer in all the experiments was optimized with BSA 2%, Tween 20 2%, and PBS 10 mM, at pH 7.4. The concentration *E. coli* O157 bacteria used at this stage to compare the intensity in the different concentration of *E. coli* detection antibody was 2×10^7^ (CFU mL^−1^). By applying sandwich LFIA, the result in Fig. S3 indicated there is no difference in signal intensity at concentrations higher than 6 μg mL^−1^ of *E. coli* detection antibody on the test-line, and this value was selected.

**Fig. 3:**
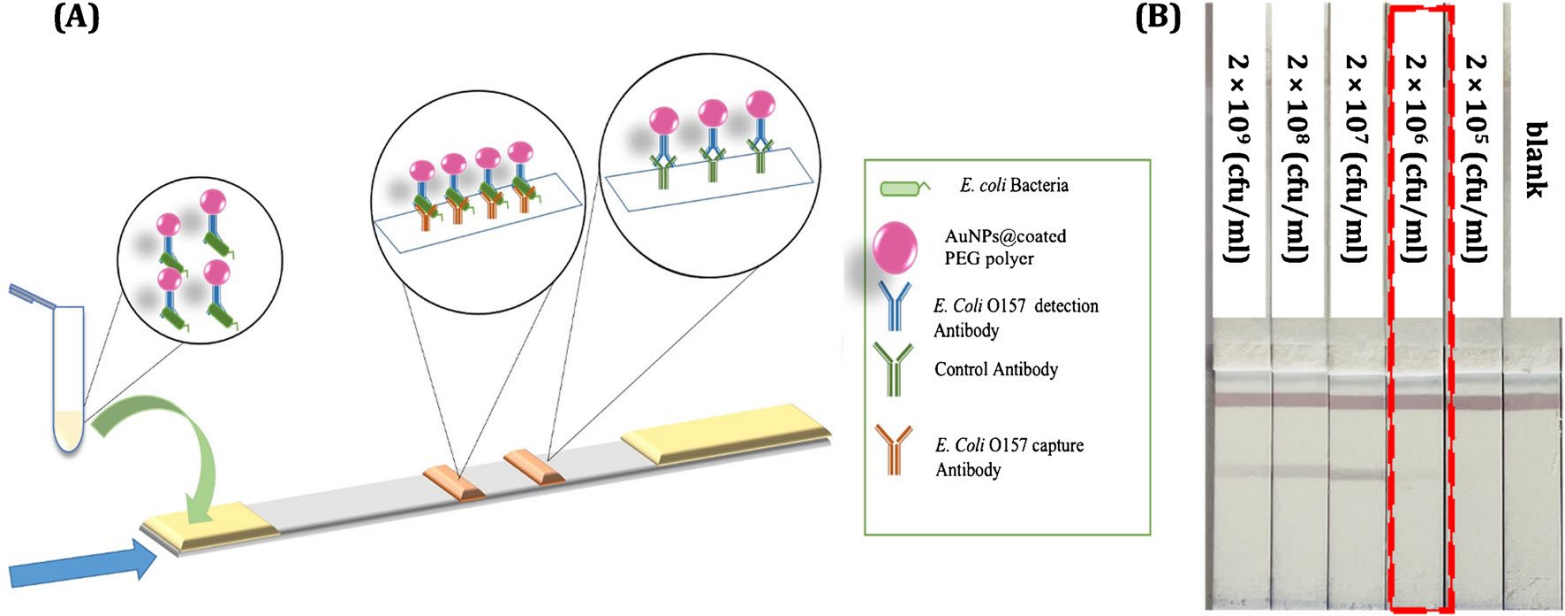
**A** Schematic illustration of the sandwich LFIA test, **B** strips in presence of different concentration of *E. coli* O157. In panel **B**, the bottom line is the test line, and the upper line, the control line

Then, a serial dilution of *E. coli* O157 bacteria starting at the initial concentration of 2×10^9^(CFU mL^−1^) was prepared in 10 mM PBS pH 7.4. The sandwich LFIA was carried out in dipstick format. For the first step, 10 μl of complex of AuNPs-*E. coli* O157 Ab (0.2 mg mL^−1^) and 25 μl of different concentrations of standard solution of *E. coli* O157 bacteria were transferred into a microtube. Then, running buffer was added with final volume of 100 μL. The components of running buffer included BSA (2%), Tween 20 (2%), and PBS (10 mM), at pH 7.4. After mixing, the solution was incubated for 15 min and then, the strips were immersed in it and the immunochromatography was carried out in dipstick format for 15 min. After this time, the strips were washed with Mili-Q water to remove extra nanoparticles and avoiding a high background at silver enhancement. Figure 3 A and B show the schematic of sandwich LFIA and the strips in the presence of different concentration of standard solution of *E. coli* O157:H7 bacteria. The first line from the bottom is the test line, where capture *E. coli* O157:H7 antibody was immobilized by physical adsorption. The immunochromatography was performed in all the experiments for 15 min and then the strips were washed with Mili-Q water. The minimum concentration that could be detected with the naked eye was 2×10^6^ (CFU mL^−1^). Fig. S4 shows the results of repeatability of the assay with different batches of AuNPs conjugated with *E. coli* O157: H7 detection antibody in response to different concentrations of *E. coli* O157:H7 at lower concentration of 2×10^6^ (CFU mL^−1^) before applying the silver enhancement procedure. At concentration lower than 2×10^6^ (CFU mL^−1^), it was not possible to detect any line visually. A silver enhancement protocol was developed for that concentration range and combined with LFIA.

### Silver enhancement for E. coli O157 bacteria detection

**Fig. 4:**
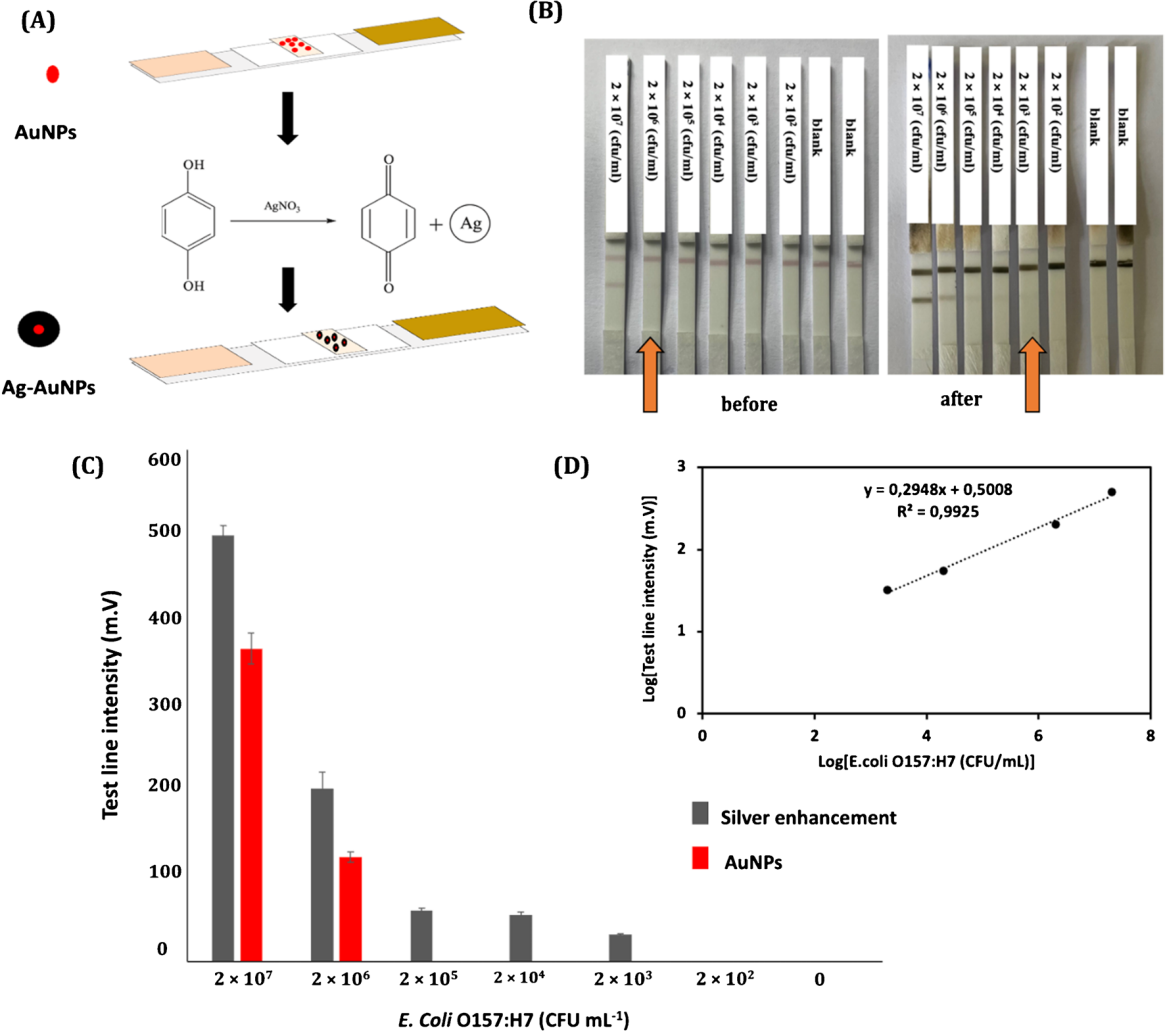
**A** Schematics of the silver enhancement method and its effect on LFIA strips in the presence of various concentrations of *E. coli* O157:H7; **B** The minimum detectable concentration with AuNPs was 2 × 10^6^ (CFU mL^−1^) and it dropped to 2 × 10^3^ (CFU mL^−1^) after silver enhancement; **C** Signal intensities obtained for different concentrations of *E. coli* O157:H7 using AuNPs (red bars) an after silver enhancement (gray bars). The graph shows the mean ± SD of three independent measurements. **D** The calibration plot using the developed LFIA in the presence of different concentration of *E. coli* O157:H7 after silver deposition for 4 min

The silver enhancement strategy is based on the catalytic reduction of silver ions on the surface of the AuNPs, which changes the color of the line from red to black (Fig. 4A). Our protocol was based on the immersion method, as explain in the previous section. The reaction time of silver deposition was optimized over by preparing a series of strips from 2×10^6^ (CFU mL^−1^) to 2×10^3^ (CFU mL^−1^). After silver deposition, the signal of false positive on the blank strips began to appear after 5 min, as shown in Fig. S5 A. Therefore, the ideal reaction time was determined to be 4 min, but longer times resulted in increased color intensity at that line.

A series of strips with different concentration of bacteria between 2×10^2^ (CFU mL^−1^) to 2×10^6^ (CFU mL^−1^) was prepared and the silver enhancement was performed following a 4-min silver deposition process (Fig. 4B). After silver enhancement, the visual LOD for *E. coli* bacteria decreased to 2×10^3^ CFU mL^−1^. This led to a threefold improvement in sensitivity compared to the conventional LFIA system. Figure 4 C shows a comparison of the results obtained for the *E. coli* O157:H7 detection before and after silver enhancement. The findings of the experiments conducted using different batches were evaluated for their repeatability, and the results are illustrated in Fig. S5 (B and C). A portable strip reader was used to quantify the color intensity of the test line in different concentration by reflectance measurements after silver enhancement. Based on the generated signals, the linear relationship was established between 2 × 10^3^ and 2 × 10^6^ (CFU mL^−1^), and the linear regression equation was obtained for the intensity (I) and the bacteria concentration (CFU mL^−1^): Log I (m.V) = 0.2948 Log [*E. coli*] (CFU mL^−1^) + 0.5008, R^2^=0.9925 (Fig. 4D). The LOD with the instrumental reader was 11 (CFU mL^−1^). It was calculated by using the (3 ×σ) / S) criterium, where σ is the standard deviation of the y-intercept and *S* is the slope of the calibration curve (*S*).

The coefficient of variation of the method (CV%) was between the range of 2.2 to 9.8% for different concentration of bacteria, which demonstrates acceptable precision for a rapid method [38].

Compared to previously published studies regarding the improvement of the sensitivity of the conventional LFIA (Table 1), AuNPs coated with the heterobifunctional PEG polymer with carboxyl groups followed by the silver enhancement has several advantages. Firstly, the use of functionalized AuNPs allows for greater control over the orientation and density of the antibodies on the surface of the nanoparticles. This can improve the specificity and sensitivity of the LFIA, as well as reduce non-specific binding of other proteins or biomolecules in the sample. Secondly, the functionalized AuNPs can also improve the stability and shelf-life of the LFIA, as the surface coating can prevent aggregation or denaturation of the antibodies and maintain their activity for longer periods of time [39]. Thirdly, the carboxyl groups on the surface of the functionalized AuNPs can also be used for conjugation with other molecules, such as enzymes or fluorescent dyes, which can further enhance the detection and visualization of the target analyte [40, 41]. Compared to magnetic nanoparticles, heterobifunctional AuNPs are more consistent in size and shape, more stable, and more adaptable for creating assays for diverse purposes [42]. Moreover, compared to other techniques like fluorescence microscopy that require specialized equipment and costly fluorescent dyes, silver enhancement offers a more accessible and cost-effective approach for signal amplification and detection. Silver enhancement does not require specialized imaging equipment to detect the signal, and the silver-enhanced lines or dots can be visualized with the naked eye or using a simple benchtop scanner. This makes silver enhancement a more accessible option for point-of-care testing or field use, especially in resource-limited settings where access to specialized equipment and expensive reagents may be limited [43].

**Table 1:** Comparison of the LOD of the developed technique with the other developed LFIA for detection of pathogens

Target bacteria	Type of label	Visual LOD (CFU mL^−1^)	Quantitative LOD (CFU mL^−1^)	Ref.
*E. coli* O157:H7	Fluorescent microsphere	3 × 10^3^	–	[44]
*E. coli* O157:H7	AuNPs	7.8 × 10^5^ (water) 3 × 10^6^ (milk)	–	[5]
*E. coli* O157:H7	AuNPs	1.87 × 10^4^	–	[45]
*E. coli* O157:H7	Au@AgNPs	5 × 10^4^	–	[46]
*B. cereus*	AuNPs	10^4^	–	[47]
*Xanthomonas arboricola pv. pruni i*	Carbon NPs	10^4^	–	[48]
*S. typhimurium*	Fluorescent-magnetic nanospheres (CFMNs)	3.75 × 10^3^	3.5 × 10^3^	[49]
*E. coli* O157:H7	Magnetic nanoparticles	10^6^	6.2 × 10^4^	[50]
*E. coli* O157:H7	Au@AgNPs	2 × 10^3^ (PBS buffer) 2 × 10^3^ (tap water and orange juice) 2 × 10^2^ (liquid milk)	1,1 x 10	This work

In terms of the selection technique for silver deposition, the immersion protocol is a more simple, cost-effective, and adaptable method. It involves immersing the test strip in a microtube containing a mixture of silver nitrate and hydroquinone/citrate buffer. In contrast, the other techniques require the preparation of glass fibers separately which can increase the risk of false positives in the results due to the sensitivity of silver nitrate to light [14].

**Fig. 5:**
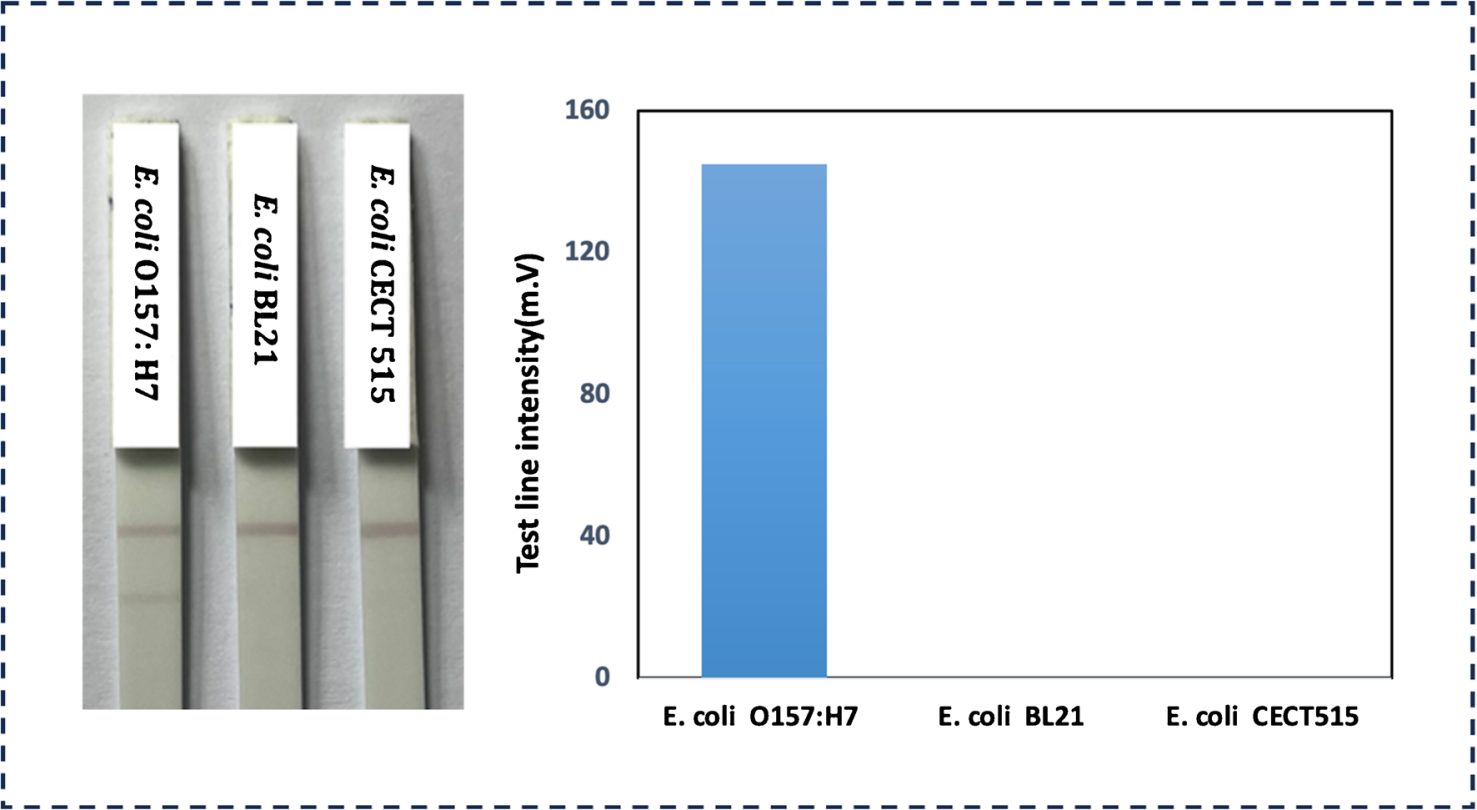
Specificity and selectivity research results against non-target bacteria *E. coli* BL21 and *E. coli* CEC T515 with a concentration of 10^10^ (CFU mL^−1^)

Furthermore, the specificity and selectivity of the newly developed assay was tested against non-target bacteria *E. coli* BL21 and *E. coli* CEC T515 (at a concentration of 10^10^ CFU mL^−1^). The results in Fig. 5 reveal that the current intensity towards *E. coli* O157:H7 is significantly higher compared to the intensity obtained for the analysis of the other bacteria, indicating that the system exhibits good selectivity for this particular serotype.

Therefore, this method has advantages in comparison with the other conventional techniques for the detection of *E. coli* O157:H7, with sensitivity, and reliability for on-site applications. Moreover, it is very simple and low cost, and can be completed within 30 min without complex handling procedures, expensive instruments, and specialized staff.

### Food sample analysis

An assessment was conducted to evaluate the accuracy of the immunoassay in detecting bacteria in food samples. To determine the capability of the proposed assay to quickly detection of *E. coli* O157:H7 within 30 min, three samples (tap water, orange juice, and liquid milk) were selected for testing and spiked with the concentration of bacteria that were not visible to the naked eye (2 × 10^2^, 2 × 10^3^, and 2 × 10^5^ CFU mL^−1^). The samples (liquid milk and orange juice) were diluted as explained in the section “Food sample analysis” and spiked with *E. coli* O157:H7.

**Fig. 6:**
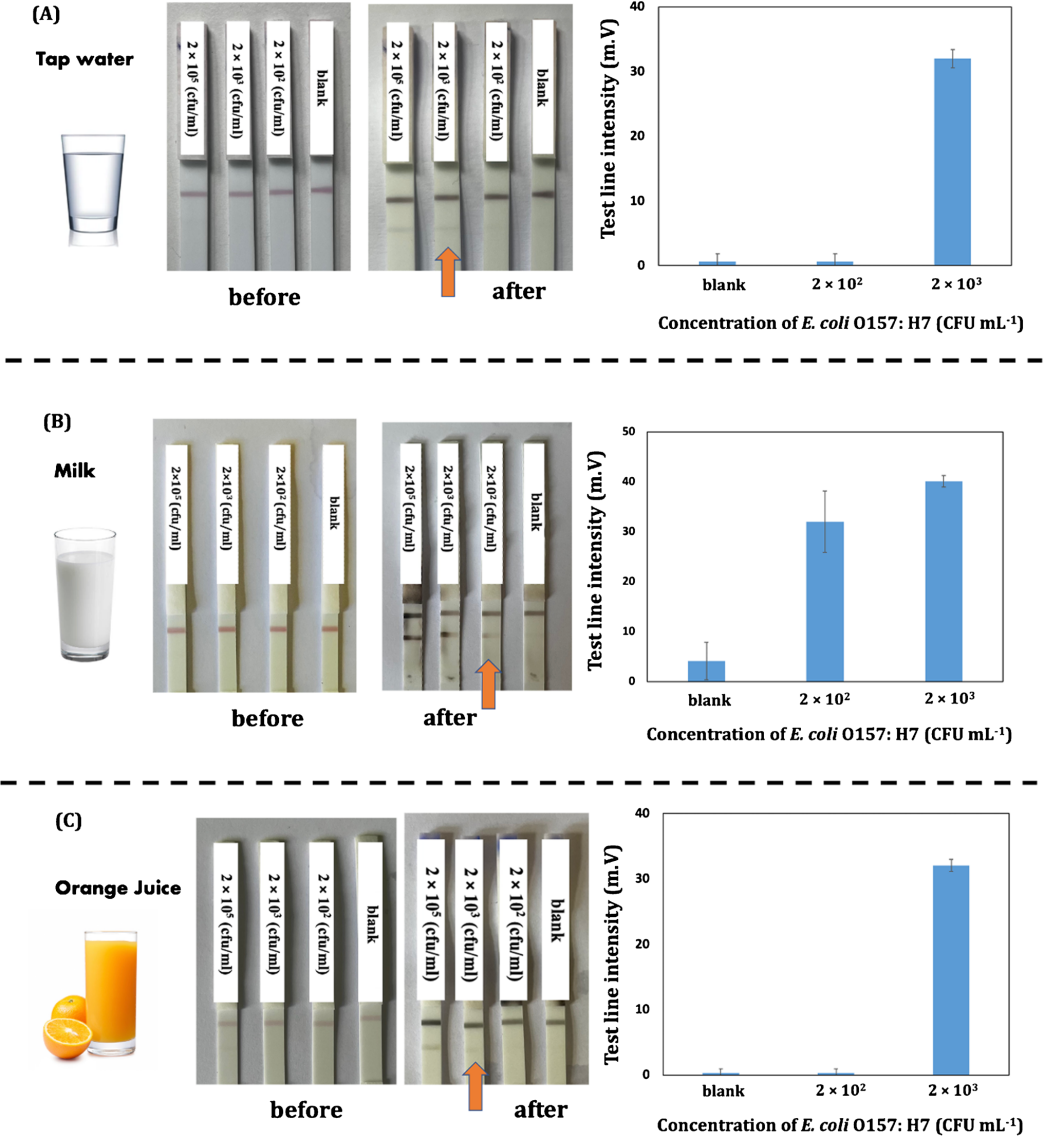
Results of the detection of *E. coli* O157:H7 in food samples by the developed assay after silver enhancement: **A** tap water, **B** milk, **C** orange juice

Figure 6 demonstrates that this newly developed assay could detect *E. coli* O157:H7 at concentrations as low as 2 × 10^2^ (CFU mL^−1^) in milk and 2 × 10^3^ (CFU mL^−1^) in tap water and orange juice. To check the reproducibility of the results, three replicates of the tests were carried out, as shown in Fig. S6. The accuracy of the developed assay was assessed by using the recovery rate of spiked samples with varying concentrations of *E. coli* O157:H7. Table 2 shows the recovery rates for each concentration. The results show a possible systematic excess error. This could be related to data following a log-normal distribution, where the most probable value is lower than the mean in a normal distribution. In this case, we had to assume a normal distribution, since we work with small series of data. Anyway, the extent of the deviation seems to be highly dependent on the matrix of the sample, being lower for tap water than for orange juice and milk, at the same level of concentration. It seems therefore that there could be an effect related to the viscosity of the sample. This could cause accumulation of errors during the dilution step, since the volumes were taken by conventional micropipettes which are designed for non-viscuous aqueous solutions. Nevertheless, the excess error in this method would protect the consumer in all cases.

**Table 2:** Recovery results obtained in the analysis of E. coli O157:H7 on spiked samples, and the respective coefficient of variation (%). Dilution factor of 1:10 was applied to the food sample used to perform the recovery study

Dilution factor	Food sample	[*E. coli*] spiked (CFU mL^−1^)	[*E. coli*] detected (CFU mL^−1^)	Recovery (%)	CV (%)
1:10	Liquid milk	0	< LOD		
		2×10^1^	< LOD		
		2×10^2^	2.5 ×10^2^	125	8.3
		2×10^3^	3.2 ×10^3^	158	5.7
1:10	Orange juice	0	< LOD		
		2×10^1^	< LOD		
		2×10^2^	< LOD		
		2×10^3^	3 ×10^3^	149	1.8
-	Tap water	0	< LOD		
		2×10^1^	< LOD		
		2×10^2^	< LOD		
		2×10^3^	2.5 ×10^3^	125	4.4

Recovery = [(Detected concentration/Spiked concentration)] × 100.

On the other hand, the reproducibility of the method was good, with coefficients of variation (CV%) lower than 2% for orange juice, lower than 5% for tap water, and lower than 9% for the milk sample. These values are lower than those of other LFIAs reported in the literature [46]. The CV was also calculated assuming a normal distribution (the median and the mean of the small series of data were very close). The values at Table 2 show that the CV is also dependent on the type of sample, and higher for the lower concentration detected, as expected when results are close to the LOD. The observed difference at the ability of the developed LFIA assay to detect low concentrations of *E. coli* O157:H7 in milk, down to 2 × 10^2^ CFU mL^−1^ could be attributed to the different composition of each matrix. Milk is a complex biological fluid with high protein and fat content, whereas orange juice and tap water are less complex matrices with lower protein and fat content. In another work from our research group using biological samples, better detectability was also found when moving from standard solutions to serum samples, due to a similar effect of the higher chemical complexity of the biofluid matrix [50].

The results demonstrate the effectiveness of the developed LFIA in detecting *E. coli* O157:H7 in various food samples.

## Conclusion

In conclusion, this study has introduced a highly sensitive LFIA combined with a silver enhancement strategy that allows for rapid detection of *E. coli* O157:H7. This approach provides several benefits over other LFIA methods that have been previously reported in the literature for this system. The assay is rapid and can provide results in 30 minutes. The use of heterobifunctional PEG polymer AuNPs with carboxyl groups makes the system more adaptable for detecting different target analytes, improving its stability and shelf-life, specific, and minimize the non-specific binding, which ensures reliable results. Silver enhancement technique was able to assess a linear relation ranging between 2 × 10^3^ (CFU mL^−1^) and 2 × 10^6^ (CFU mL^−1^), and it dropped the LOD to 2 × 10^3^ (CFU mL^−1^). The assay’s specificity, selectivity, and accuracy were tested against non-target bacteria and actual food samples, with promising outcomes. Recovery analysis showed that the method can efficiently detect at least 2 × 10^2^ (CFU mL^−1^) of *E. coli* O157:H7 in milk samples and 2 × 10^3^ (CFU mL^−1^) in orange juice and tap water, with good recoveries and CV (< 10%) in all cases tested. Therefore, compared to other LFIAs reported in the literature, this method is highly specific, rapid, low-cost, and reliable to protect the consumer. These characteristics could make this method a good candidate for the rapid detection of *E. coli* O157:H7 bacteria and other pathogens in industry and medical fields.

## Supplementary Information

Below is the link to the electronic supplementary material.

ESM 1 (PNG 291 kb)

High resolution image (TIFF 539 KB)

ESM 2 (PNG 1.16 mb)

High resolution image (TIF mb)

ESM 3 (PNG 839 kb)

High resolution image (TIFF 4.22 MB)

ESM 4 (PNG 1.67 mb)

High resolution image (TIFF 1.21 MB)

ESM 5 (PNG 4.69 mb)

High resolution image (TIFF 3.06 KB)

ESM 6 (PNG 1.08 mb)

High resolution image (TIFF 785 KB)
