# Supplementary figures and images for: Rapid and sensitive detection of E. coli O157:H7 by lateral flow immunoassay and silver enhancement
Source: Mikrochim Acta. 2023 Jun 19;190(7):264. doi: 10.1007/s00604-023-05834-8 (PMC10279566; doi:10.1007/s00604-023-05834-8)

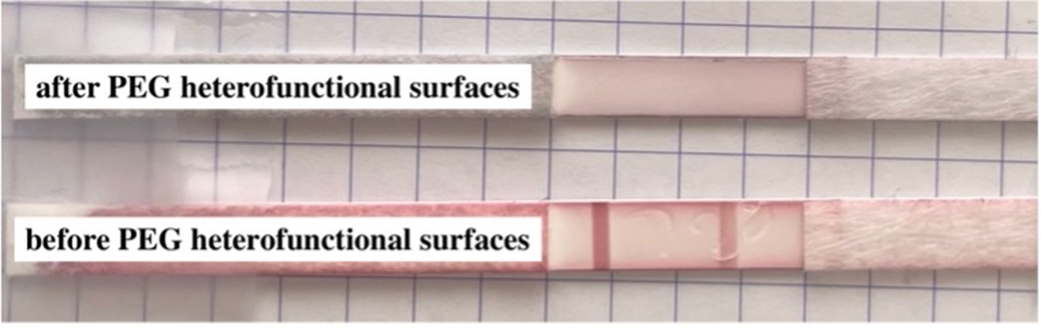

Supplement: Supplementary file 1 — ESM 1 (PNG 291 kb) [file 604_2023_5834_Fig6_ESM.png]

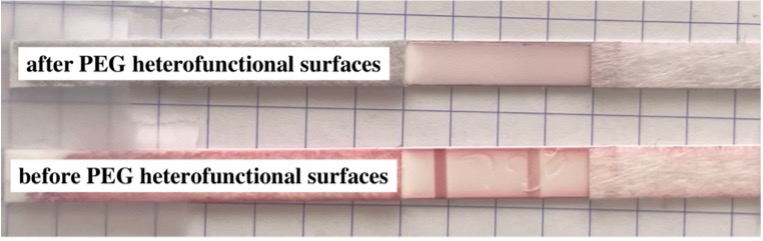

Supplement: Supplementary file 2 — High resolution image (TIFF 539 KB) [file 604_2023_5834_MOESM1_ESM.tiff]

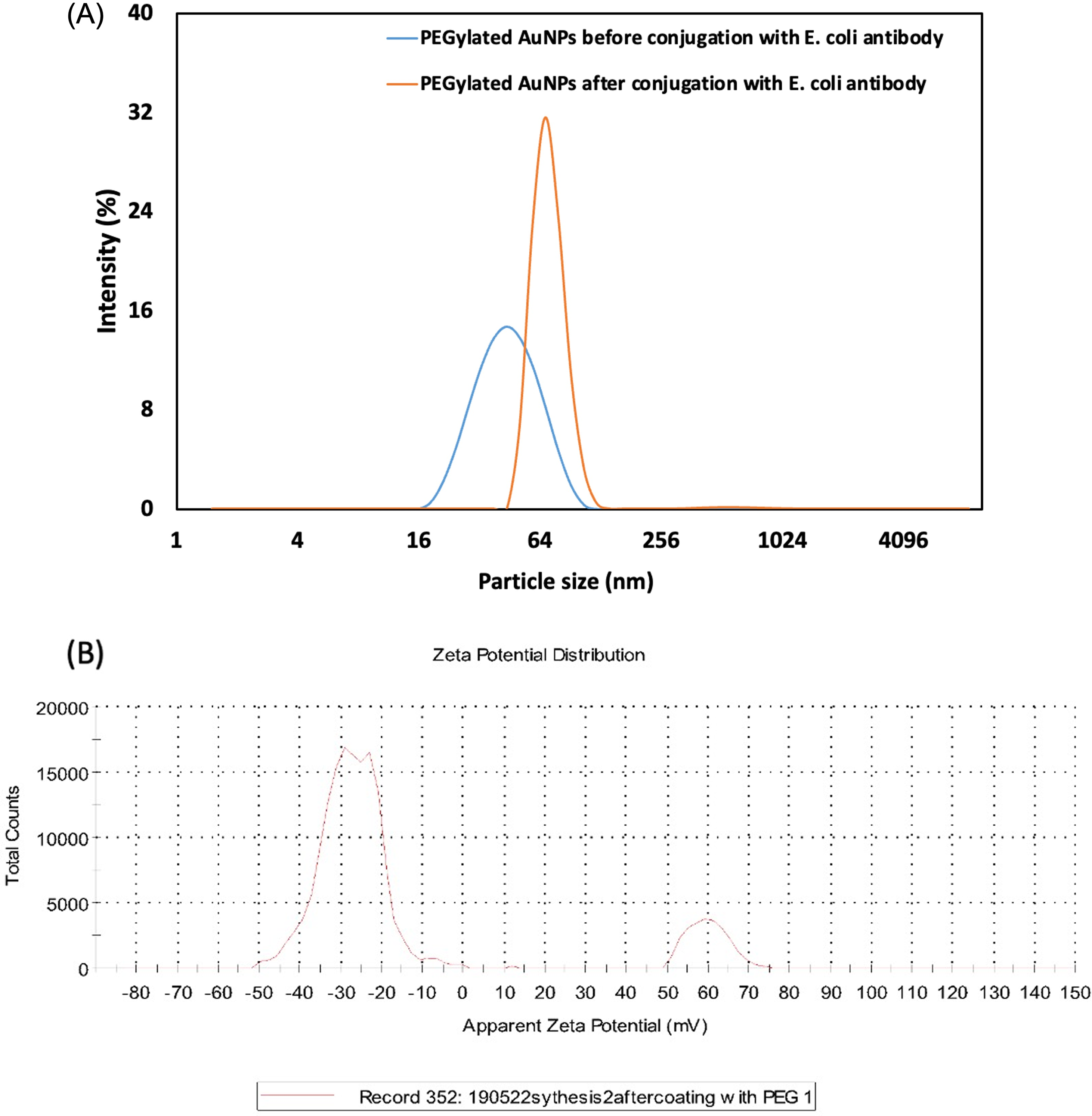

Supplement: Supplementary file 3 — ESM 2 (PNG 1.16 mb) [file 604_2023_5834_Fig7_ESM.png]

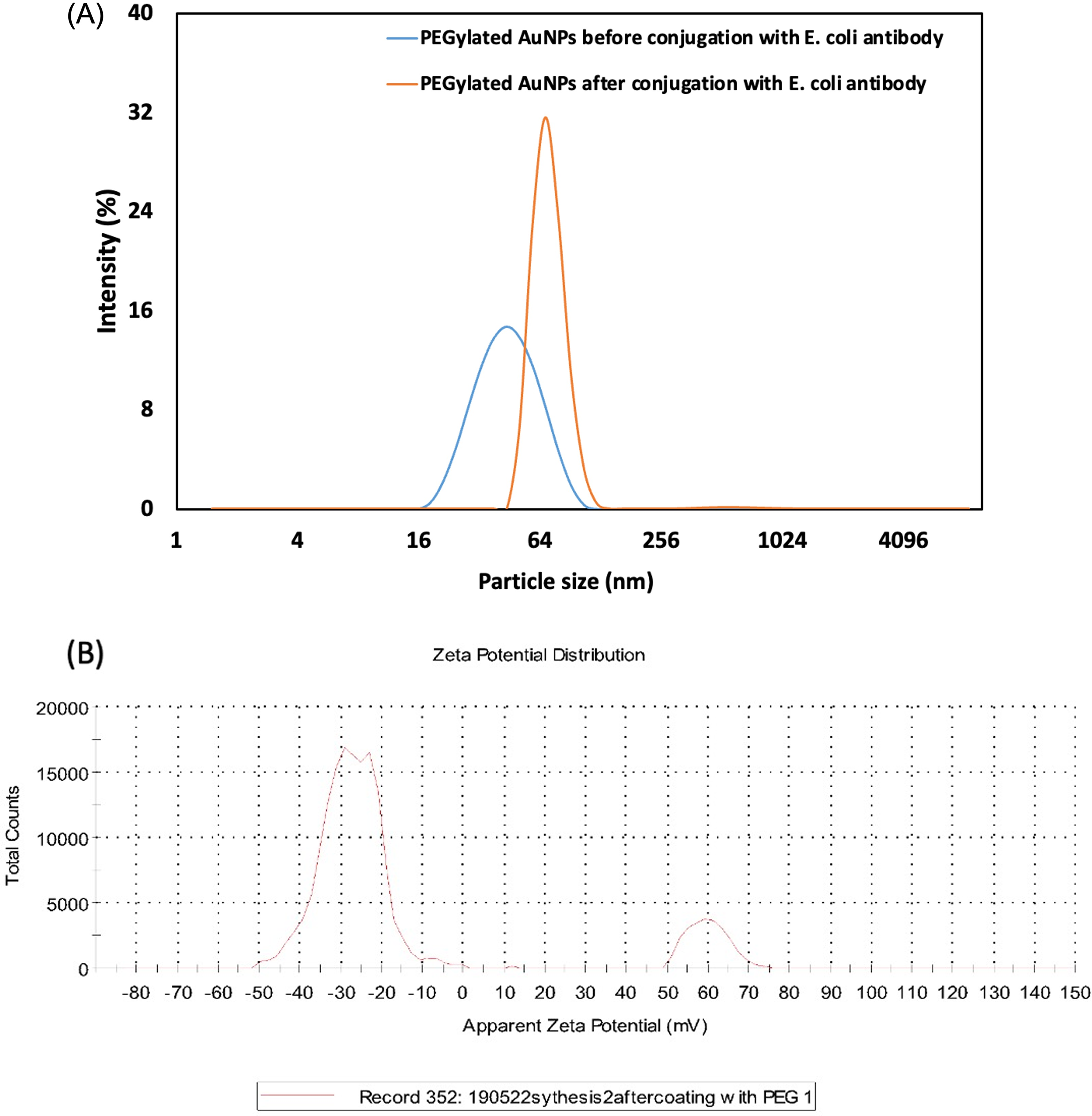

Supplement: Supplementary file 4 — High resolution image (TIF mb) [file 604_2023_5834_MOESM2_ESM.tif]

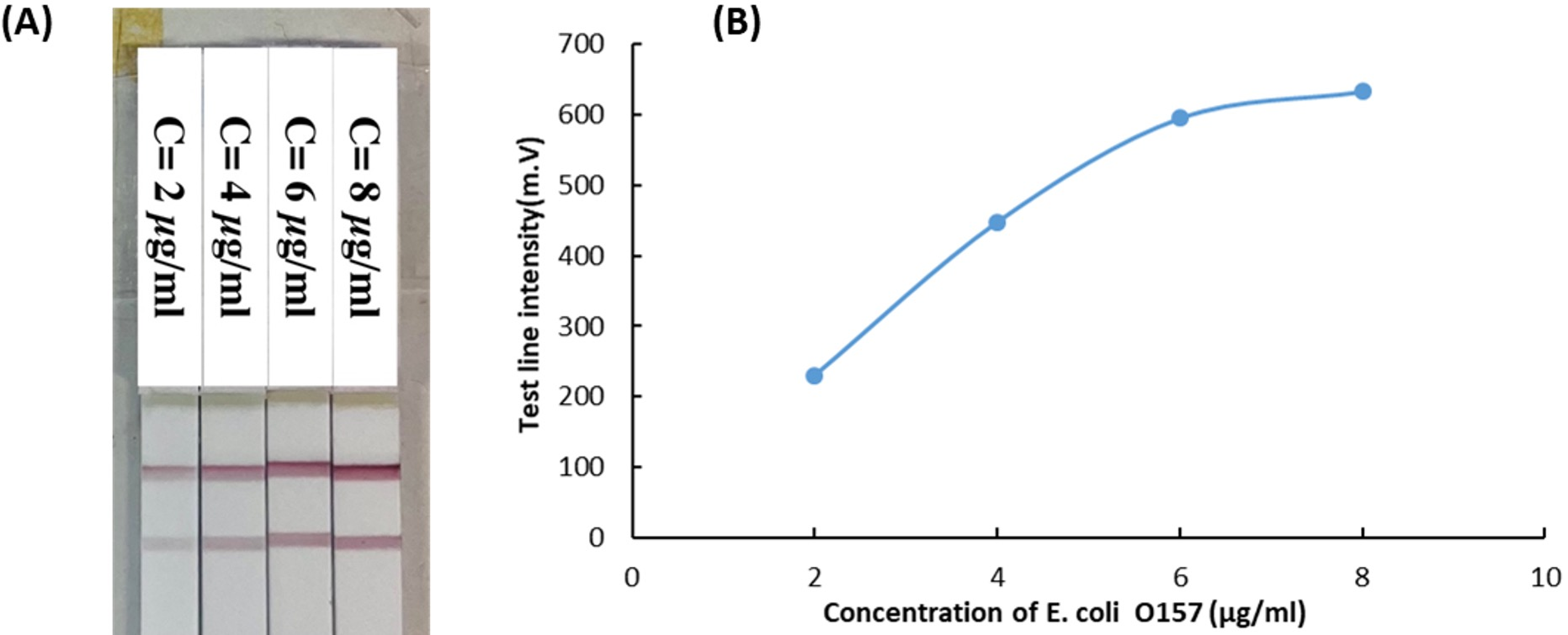

Supplement: Supplementary file 5 — ESM 3 (PNG 839 kb) [file 604_2023_5834_Fig8_ESM.png]

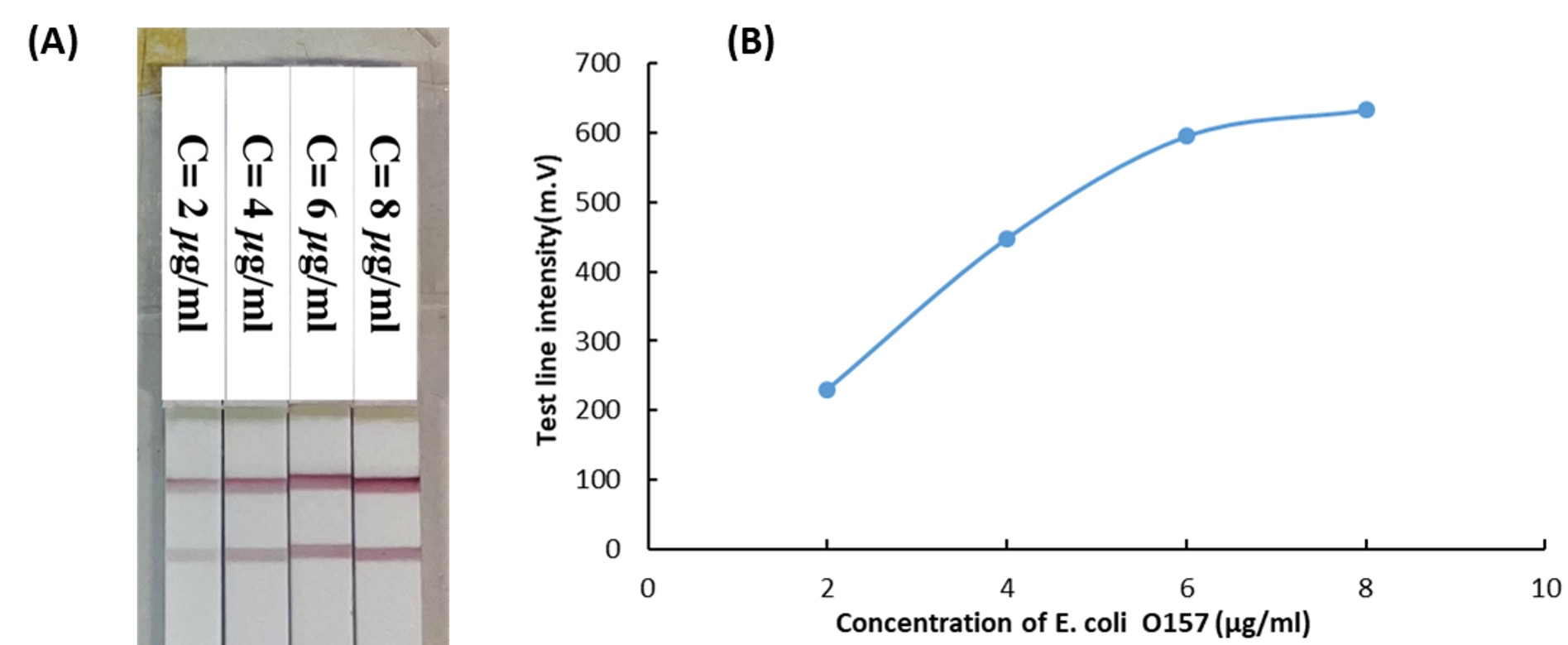

Supplement: Supplementary file 6 — High resolution image (TIFF 4.22 MB) [file 604_2023_5834_MOESM3_ESM.tiff]

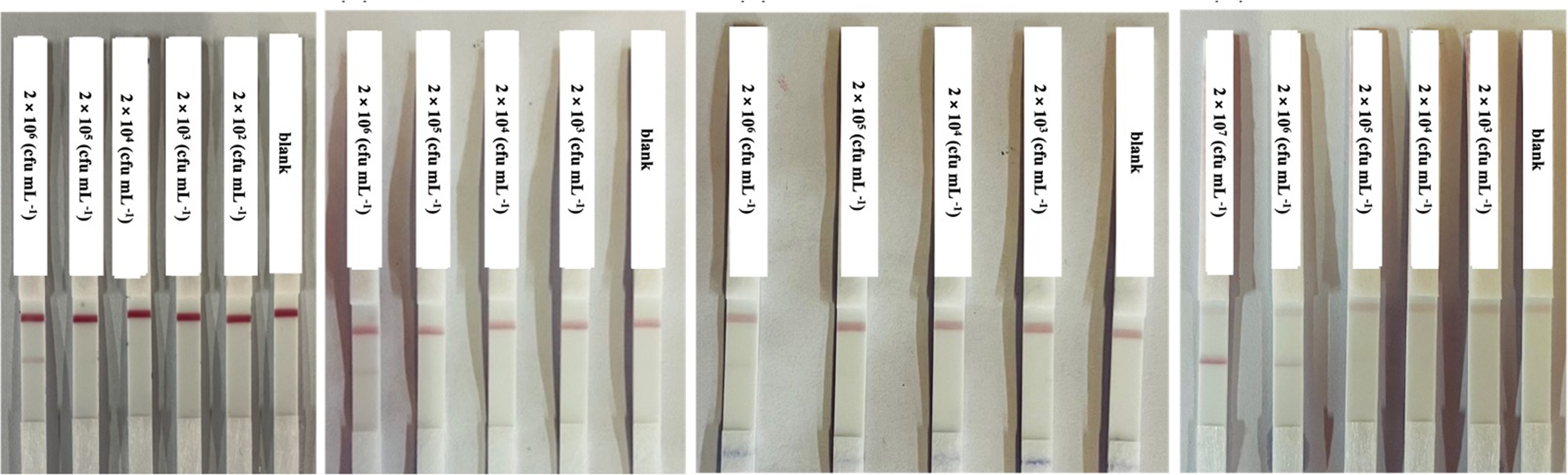

Supplement: Supplementary file 7 — ESM 4 (PNG 1.67 mb) [file 604_2023_5834_Fig9_ESM.png]

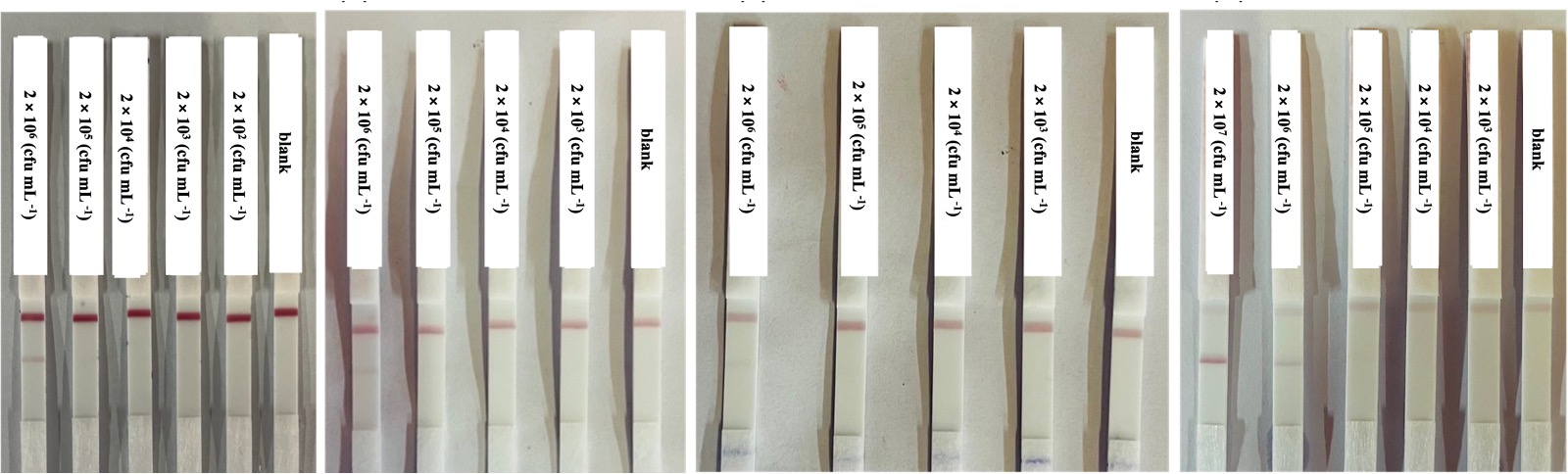

Supplement: Supplementary file 8 — High resolution image (TIFF 1.21 MB) [file 604_2023_5834_MOESM4_ESM.tiff]

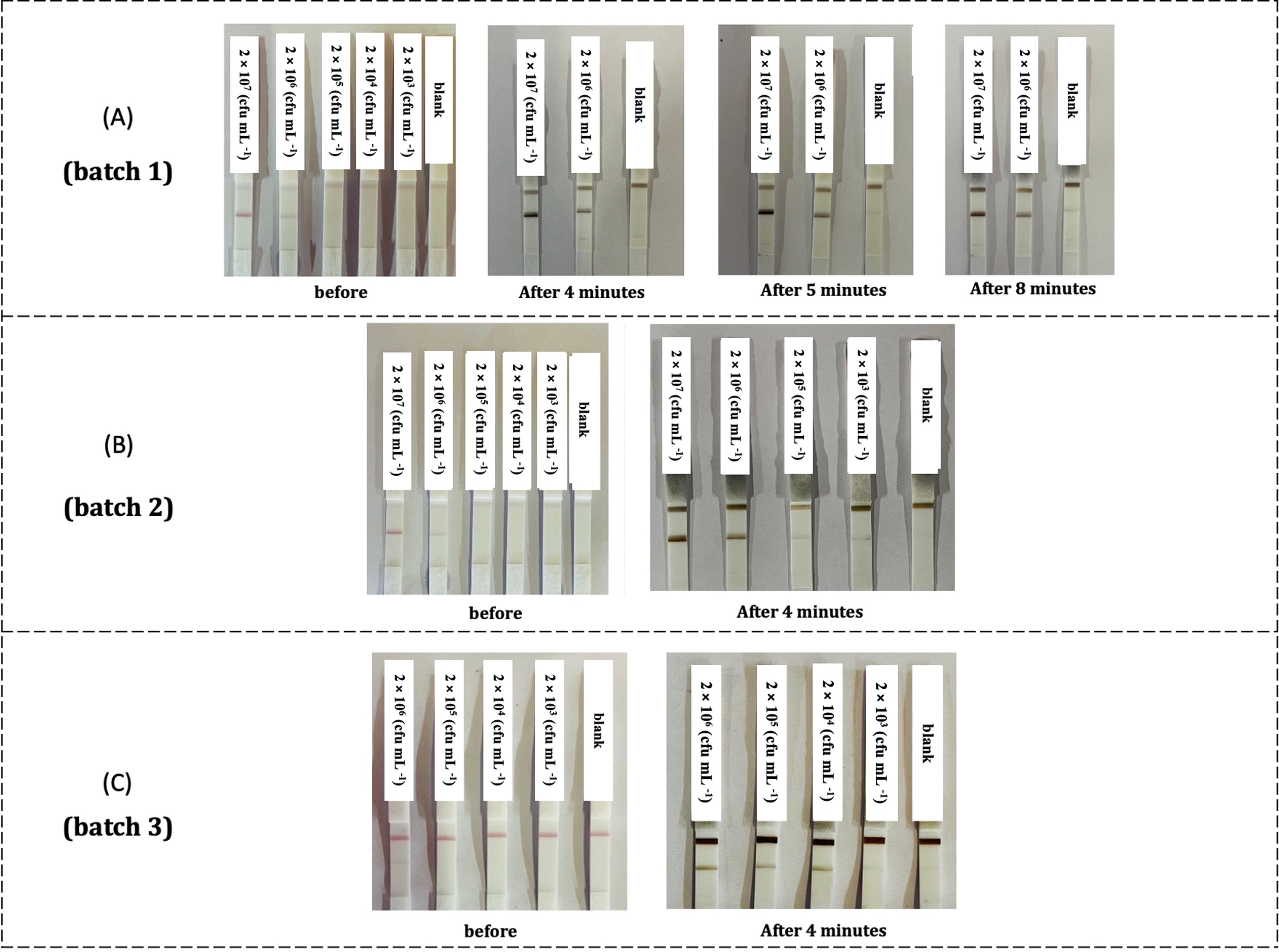

Supplement: Supplementary file 9 — ESM 5 (PNG 4.69 mb) [file 604_2023_5834_Fig10_ESM.png]

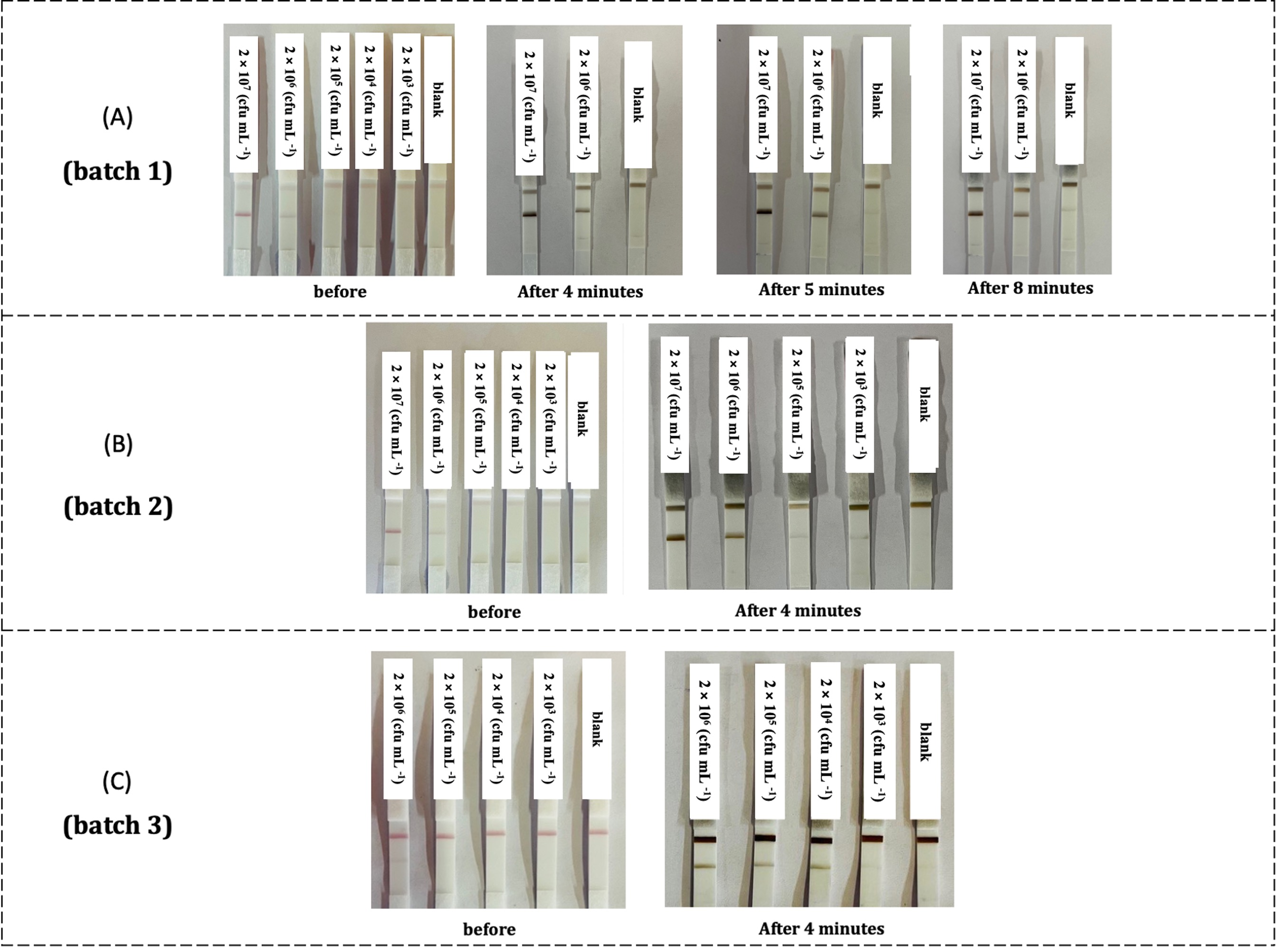

Supplement: Supplementary file 10 — High resolution image (TIFF 3.06 KB) [file 604_2023_5834_MOESM5_ESM.tiff]

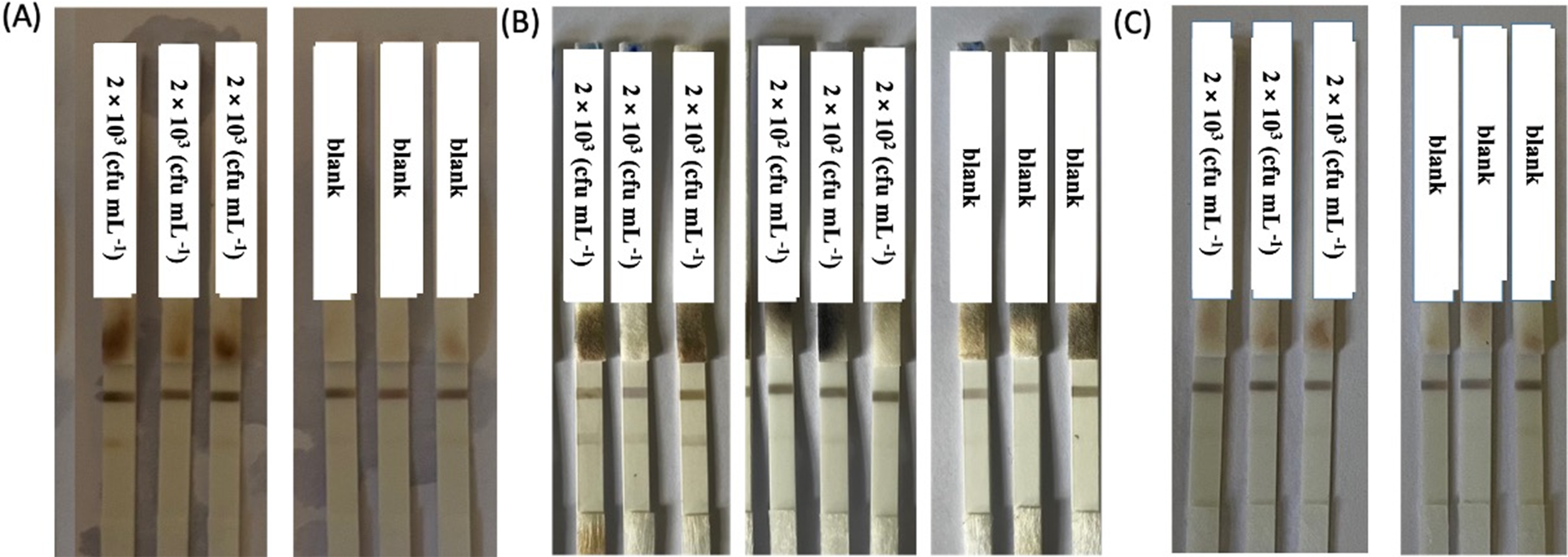

Supplement: Supplementary file 11 — ESM 6 (PNG 1.08 mb) [file 604_2023_5834_Fig11_ESM.png]

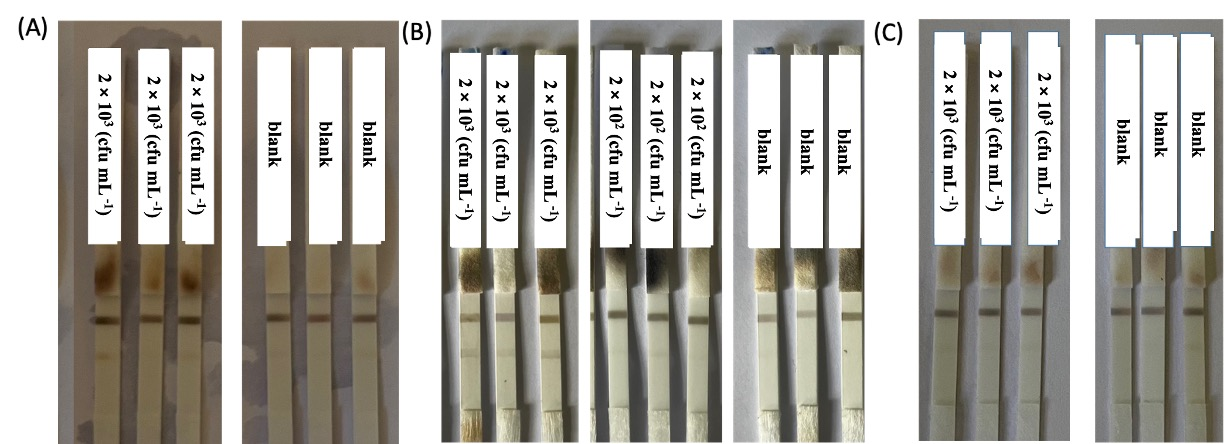

Supplement: Supplementary file 12 — High resolution image (TIFF 785 KB) [file 604_2023_5834_MOESM6_ESM.tiff]
